# Perianal Mucinous Adenocarcinoma: A Case of Recurrent Anal Fistula

**DOI:** 10.7759/cureus.58795

**Published:** 2024-04-22

**Authors:** Zhuoneng Chen, Chaohui Yu

**Affiliations:** 1 Department of Gastroenterology, The First Affiliated Hospital, Zhejiang University School of Medicine, Hangzhou, CHN

**Keywords:** perianal mucinous adenocarcinoma, crohn’s disease (cd), perianal disease, complex anal fistula, mucinous adenocarcinoma

## Abstract

An anal fistula is a fairly common clinical condition, with a very low incidence of malignant transformation. Mucinous adenocarcinoma is a subtype of adenocarcinoma, and its occurrence within perianal fistula tracts is quite rare. This case report describes a 54-year-old male patient with recurrent anal fistula, initially suspected of Crohn's disease (CD), and ultimately diagnosed with perianal mucinous adenocarcinoma. After our joint internal medicine, surgery, and imaging reassessment, the diagnosis was confirmed. Anal fistula is usually considered a benign lesion, but it may also be associated with other diseases. Due to overlapping symptoms of related diseases, the investigation of malignant lesions is often overlooked. This case report emphasizes the importance of timely referral and multidisciplinary management for disease diagnosis and early treatment.

## Introduction

An anal fistula is an abnormal channel that connects the inner wall of the rectum to the skin or peripheral tissue around the anus. It is usually caused by infections or other diseases around the anus, including but not limited to perianal abscess, perianal inflammation, and Crohn's disease (CD) [1]. Malignant transformation of a perianal fistula is not common. The overall incidence of fistula-associated anal cancer or cancer arising from perianal fistulas is 0.3-0.7% [2,3]. In most cases, prolonged chronic inflammation is a possible cause of fistula-associated cancer [4].

Mucinous adenocarcinoma is a unique subtype of colorectal cancer (CRC). This kind of adenocarcinoma is distinguished by its prominent production of mucin, a gel-like substance found in the extracellular matrix. In this subtype, mucin accounts for at least 50% of the tumor's volume, imparting a characteristic appearance of abundant mucin pools within the tumor tissue [5]. Relevant statistical data show that 10-20% of CRC patients belong to mucinous adenocarcinoma, and mucinous adenocarcinoma is more common in women and young patients [6]. In addition, mucinous colorectal adenocarcinoma is more common in the proximal colon than in the rectum or distal colon [7]. Due to the lack of specific symptom manifestations, diagnosis is frequently delayed.

Therefore, we reported a case of mucinous adenocarcinoma in the perianal fistula of a patient with a recurrent anal fistula. This case emphasized the importance of multidisciplinary approaches in internal medicine, surgery, and imaging for disease diagnosis and treatment.

## Case presentation

**Figure 1:**
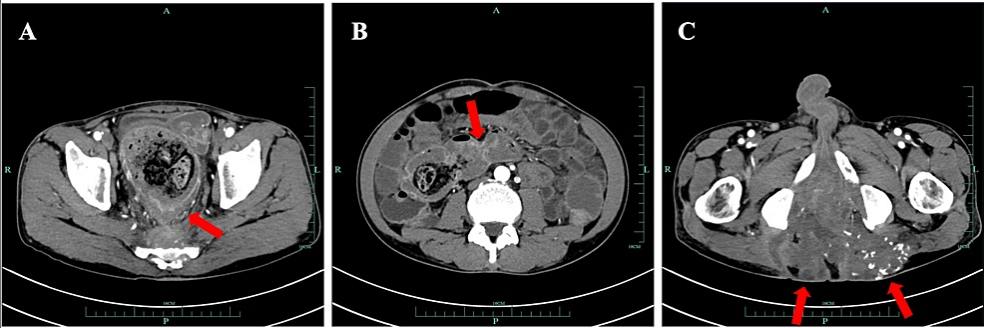
Three-dimensional computerized tomography imaging results of the small intestine and blood vessels. (A) Due to the thickening of the rectal wall and narrowing of the lumen, there is significant dilation of the colon with abundant contents (arrows). (B) The mucosal wall of the ileum shows partial circular abnormal enhancement (arrows). (C) Multiple fistulas are observed around the rectum and anal canal, with large areas of abnormal density and scattered calcifications in some regions. The lesions extend into both gluteus maximus muscles (arrows).

A 54-year-old male came to the digestive department for treatment due to recurrent diarrhea. The patient has had recurrent diarrhea, accompanied by abdominal distension, weight loss, and occasional abdominal pain in the past 10 years. During this period, multiple anal fistula surgeries were performed at other hospitals. Recently, the patient has had frequent diarrhea, five to six times per day, presenting as yellow, loose, or watery stools, accompanied by perianal pain and dull pain in the posterior lateral muscle group of the left thigh, affecting walking. He developed a fever the day before seeking medical attention. During the physical examination, it was found that the patient's perianal skin was swollen, but no obvious external opening was observed; the body mass index was 22.9 kg/m^2^. The three-dimensional computed tomography (CT) imaging results of the patient's small intestine and blood vessels indicated segmental inflammation of the ileum, the inflammatory thickening of the rectal and anal walls, narrowing and obstruction of the intestinal lumen, and secondary colon dilation and fluid accumulation. Widespread inflammation and fistula formation could be found in the lower segment of the rectum and perianal space, sacral anterior space, bilateral ischiorectal fossa, and gluteus maximus muscle space (Figure 1). Based on the patient's clinical symptoms, clinical doctors first considered the possibility of CD. Therefore, further improvements should be made in examinations, such as colonoscopy and anal fistula magnetic resonance imaging (MRI).

**Figure 2:**
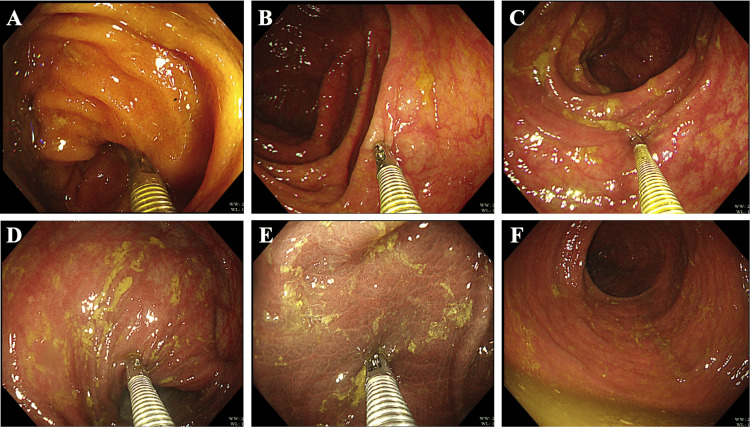
Colonoscopy examination images. (A) Terminal ileum. (B) Ascending colon. (C) Transverse colon. (D) Descending colon. (E) Sigmoid colon. (F) Rectum.

Surprisingly, the colonoscopic examination of the patient did not reveal the typical features of CD, such as obvious segmental mucosal congestion and edema, cobblestone-like paving stones, or longitudinal ulcers. Under colonoscopy, the patient's entire colonic mucosa showed black-speckled changes, and we performed biopsies in multiple locations of the colon (Figure 2). However, no crypt deformation or non-caseating granuloma was observed in the biopsy specimens from multiple locations. The pathological results only indicated mild inflammation of the intestinal mucosa, with aggregation of tissue cells that phagocytose pigments in the lamina propria. The colon lesion was consistent with changes in colonic melanosis.

**Figure 3:**
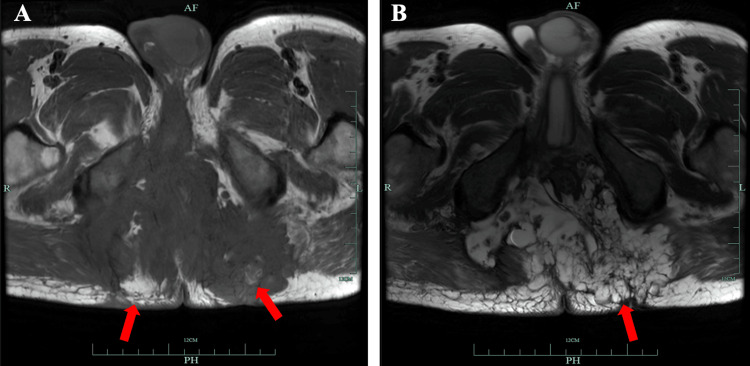
Magnetic resonance imaging of anal fistula. (A) T1-weighted images show patchy, iso-signal lesions around the anal region, with some areas exhibiting patchy high signal intensity (arrows). (B) T2-weighted images show perianal abnormal high-signal lesions. Multiple separated, well-defined, cystic lesions are predominantly seen on the left side of the subcutaneous tissue of the buttocks (arrows).

In addition, simultaneous anal fistula MRI results showed extensive inflammation and fistula formation in the lower rectal segment and the perianal space, sacral anterior space, bilateral ischiorectal fossa, and gluteus maximus space, as well as the formation of perianal abscess, presenting as a complex anal fistula (Figure 3). We asked a doctor from the colorectal surgery department to evaluate and improve the anal fistula biopsy. Three skin tissue samples were taken for histological analysis, and the pathological results indicated perianal mucinous adenocarcinoma.

## Discussion

An anal fistula is a common disease in clinical practice, but its malignant transformation is very rare. For cases of recurrent anal fistulas, we often consider CD first. CD is a chronic inflammatory disease of the gastrointestinal tract, with an increasing incidence worldwide [8]. The pathogenesis of CD is not yet fully understood and may be related to multiple factors, such as genetics, abnormal immune function, and environmental factors. It can cause lesions from the mouth to the anus and may lead to extraintestinal complications. Common clinical symptoms include abdominal pain, diarrhea, bloating, weight loss, anemia, and fatigue. Up to 25% of CD patients discover perianal fistula during the course of the disease [9,10]. CD with perianal fistula may occur before or after luminal disease, and its pathogenesis has not been fully elucidated. Most perianal fistulas appear at or after the diagnosis of CD, but 10-30% of the time, perianal fistulas may precede the diagnosis of luminal CD. Perianal CD affects 25-35% of CD patients [11]. The pathogenesis of CD perianal fistulas is not fully understood and may originate from inflammation or infection of the anal glands in CD patients or from deep penetrating ulcers in the rectum or anus [12].

Anal fistulas usually present with symptoms, such as pus discharge, painful defecation, swelling, and redness around the anus, which seriously affect the patient's quality of life. The diagnosis of chronic perianal fistula tumors is difficult because their symptoms are non-specific and often delayed. Early onset, long course (>10 years), severe chronic colitis, and chronic fistula stenosis are important risk factors for fistula cancer [13]. In patients with CD, cancer at the site of chronic perianal fistula is rare, and there is little data on its incidence rate, diagnosis, and treatment [14]. A meta-analysis of 20 clinical studies, encompassing over 40,000 CD patients, revealed that the incidence of perianal cancer caused by CD fistulas was exceedingly low, at 0.2 per 1000 patient-years [15]. Individual reports have described sporadic cases of perianal cancer in CD, most of which were histologically classified as squamous cell carcinoma or adenocarcinoma [16].

At the first visit, the patient exhibited symptoms similar to CD, such as diarrhea, bloating, weight loss, and recurrent anal fistula. Considering segmental inflammation of the ileum as indicated by small intestine CT, clinical doctors often consider CD first. However, after re-evaluating the patient, we found that the diagnostic basis for CD was insufficient. The patient was diagnosed with perianal mucinous adenocarcinoma through a timely pathological biopsy of anal tissues by the colorectal surgeon, and a timely referral was made.

Mucous adenocarcinoma is a unique subtype of adenocarcinoma characterized by a high proportion of lymph node infiltration and peritoneal implantation. It often occurs in the proximal colon [5]. Organizational analysis is the gold standard for its diagnosis. Mucous adenocarcinoma is usually associated with benign inflammation, such as chronic anal fistula, perianal abscess, diabetes, and CD [17]. Thus far, the prognostic of mucinous colorectal adenocarcinoma remains uncertain, considering factors, such as tumor location, molecular changes, population characteristics, or different treatment strategies [5]. The risk of fistula-related cancer often correlates with the duration of the disease [14]. Perianal mucinous adenocarcinoma in fistula tracts is believed to arise from dysplastic changes induced by continuous regeneration of the mucosal lining within the fistula [18]. Therefore, chronic inflammation caused by perianal fistulas should be taken seriously, prompting early medical attention, particularly in patients with atypical clinical features and recurrent anal fistulas, who should undergo early biopsy to rule out the possibility of malignancy.

The recurrent anal fistula patient with perianal mucinous adenocarcinoma is a rare disease. We emphasize the importance of early diagnosis in improving disease outcomes. The symptoms are non-specific, so we remind clinical doctors to not only consider CD for patients with recurrent anal fistulas but also be vigilant about the possibility of malignant tumors. With the help of multiple disciplines, exploration and biopsy can be carried out. Early diagnosis can lead to better results in subsequent treatment.

## Conclusions

We reported a 54-year-old male patient with a recurrent anal fistula, initially suspected of CD with a perianal fistula, and ultimately diagnosed with perianal mucinous adenocarcinoma. Due to previous doctors failing to consider malignant tumors in differential diagnosis, his cancer diagnosis was delayed. This case emphasizes the need to consider the possibility of malignant tumors in the differential diagnosis of refractory anal fistula, as well as the importance of timely referral and multidisciplinary management for disease diagnosis and early treatment intervention.
